# Treasurer’s Report for Financial Year (FY) 2021

**DOI:** 10.1093/molbev/msac244

**Published:** 2022-12-05

**Authors:** John P McCutcheon, Jeffrey L Thorne

**Affiliations:** SMBE Treasurer; SMBE Treasurer

Society’s Assets and Liabilities as of 31 December 2021:

**Table:** 

Assets	
Cash Account	2,456,446.18$
CDs	2,135,678.87$
Prepaid Expenses	138,256.98$
Accounts Receivable	875,846.00$
Total	5,606,228.03$
	
Liabilities	
Accounts Payable	25,865.58$
Deferred Membership	41,069.36$
Deferred Journal Revenue	890,656.00$
Total	957,590.94$
	
**Net Assets**	**4,648,637.09$**

This report covers the 12 months from **January 1, 2021** to **December 31, 2021**. The Society continues to be in very good financial health. For 2021, the net revenue collected by the Society was $1,553,409.96, with the main sources of revenue being our two journals, *Molecular Biology and Evolution* (*MBE*; $1,085,368) and *Genome Biology and Evolution* (*GBE*; $432,515).

In a normal year, most of the Society’s expenses are dedicated to travel for the in-person annual meeting. However, the annual meeting was again canceled in 2021 due to the COVID pandemic. The bulk of the Society’s 2021 expenses of $449,705.09 were towards payouts to journal Editors-in-Chief, journal editing and marketing efforts, the organization and implementation of the 2021 virtual meeting, and Society prizes such as the mid-career award, lifetime contribution award, and graduate student excellence awards.

This 2021 report is the first where 100% of *MBE* revenue reflects only income from Open Access fees as opposed to complete or partial revenue from a subscription model. However, the 2021 *MBE* revenue is inflated due to revenue carry-forward from 2020, resulting from a one-time shift from journal volume-based accounting to calendar-year-based accounting.

Society’s Revenues and Expenses for FY2020:

**Table:** 

**Revenues**	
Membership Dues	20,273.20$
MBE FY2020 Revenue	1,085,368.00$
MBE Profit Sharing	1,986.00$
GBE FY2020 Revenue	432,515.00$
Unrestricted Donation	1,345.00$
Interest Income	11,922.76$
***Total Revenues***	***1,553,409.96$***
	
**Expenses**	
Miscellaneous Prize & Career Awards	39,301.50$
Carer Awards	7,651.61$
*Total Prize & Awards*	*46,953.11$*
	
Operating Expenses	47,089.79$
Executive Administration	40,384.80$
Corporation Fee	40.00$
Banking Charges	1,284.12$
Tax Preparation Fees	2,000.00$
*Total Operations*	*90,798.20$*
	
MBE Editing	99,725.00$
MBE Press & Publicity	10,200.00$
GBE Editing	100,000.00$
GBE Highlight Honoraria	29,475.00$
*Total Editorial*	*239,400.00$*
	
Annual SMBE Meeting	72,553.78$
*Total Meetings*	*72,553.78$*
* *	* *
***Total Expenses***	***449,705.09$***
	
***Change in Net Assets***	*** 1,103,704.87$***

